# Rich and purposeful mathematical knowledge of mothers and children in a Torres Strait Islander community

**DOI:** 10.1186/2193-1801-3-42

**Published:** 2014-01-22

**Authors:** Bronwyn Ewing

**Affiliations:** Faculty of Education, Queensland University of Technology, Kelvin Grove Road, Kelvin Grove, 4059 Australia

**Keywords:** Torres Strait Islands, Mothers and children, Indigenous knowledge centre, Funds of knowledge, Sorting and partitioning

## Abstract

This paper focuses on a pilot study that explored the situated mathematical knowledge of mothers and children in one Torres Strait Islander community in Australia. The community encouraged parental involvement in their children’s learning and schooling. The study explored parents’ understandings of mathematics and how their children came to learn about it on the island. A funds of knowledge approach was used in the study. This approach is based on the premise that people are competent and have knowledge that has been historically and culturally accumulated into a body of knowledge and skills essential for their functioning and well-being (TIP 31:132, 1992). The participants, three adults and one child are featured in this paper. Three separate events are described with epiphanic or illuminative moments analysed to ascertain the features that enabled an understanding of the nature of the mathematical events. The study found that Indigenous ways of knowing of mathematics were deeply embedded in rich cultural practices that were tied to the community. This finding has implications for teachers of children in the early years. Where school mathematics is often presented as disembodied and isolated facts with children seeing little relevance, learning a different perspective of mathematics that is tied to the resources and practices of children’s lives and facilitated through social relationships, may go a long way towards improving the engagement of children and their parents in learning and schooling.

## Background to the project

In 2009, members of the YuMi Deadly Centre at the Queensland University of Technology were commissioned by the Torres Strait Islander Education Council (Torres Strait Islander Regional Education Council 2011) to conduct a one year project that focused on contextualising the teaching and learning of the mathematics strand of measurement (Australian Curriculum Assessment and Reporting Authority 2011) to the Islanders’ culture and home language/s in six schools from Prep to Year 7. During this process, informal conversations were held with the community, including the Head of School Campus, teachers, parents, Elders and Senior Women about working with parents and their young children (0–5). Questions about how to encourage and support parents with their children’s education with a view to preparing both parents and children for the transition to formal schooling guided this process arose. Concerns were expressed by community members about parents’ minimal involvement in their children’s formal education. One point was made that their non-involvement was because of their own negative school experiences as children. The community members realised that an education was important to future life opportunities in education, training and employment.

From those discussions, three main issues emerged. First, some community members identified with the values of Western education whereas others were still strong in their traditional cultural values. Second, community members wanted their children to be bi-culturally competent and two-way strong (Sarra et al. 2011; Overmars 2010). Finally, the mathematics used in the community would not be very different from school mathematics however, it was used in the cultural practices and language of daily life was different. The community believed that their children had the capacity to succeed like their counterparts in other urban and regional areas of Australia.

This paper elaborates the pilot project. It provides an introduction, a review of the literature to situate current understandings of Indigenous ways of knowing and community-based education drawing on Indigenous literature where possible, and the methodology that guided the project, an analysis and discussion of preliminary data and conclusion and implications.

At this point an important caveat is necessary. In this paper, the term Indigenous refers to the Aboriginal and Torres Strait Islander peoples of Australia. Even though Indigenous is a homogenising term, that is, one people one culture, the meaning of the word in the context of this paper is the opposite. We recognise and respect that Indigenous people of Australia consists of many First Nations each with their own unique culture and histories (Aboriginal and Torres Strait Island Social Justice Commissioner 2008).

## Introduction

At a time when a number of strategies have been implemented to increase Torres Strait Islander parents’ participation in education with their children, for example, reading and dance programs, going beyond the simple dichotomy between Islander parents’ ways of knowing—experience, out-of-school, intuitive, tacit and, academic—in-school, linear, deliberate is important (see for example, Department of Education, Employment and Workplace Relations 2011; Torres Strait Islander Regional Education Council 2011). For educational practice, this means inviting parents and children into a world of motivating learning where their cultural practices, ways of knowing and language are recognised, valued and respected in school. The children’s and their parents’ engagement with both the activity and the social context are fore grounded so that rich learning experiences can occur (Gonzalez et al. 2005).

The learning context becomes a site for co-sharing ideas that draws on multiple ways of knowing—funds of knowledge that are activated and tied with mathematics curricula (see for example, Australian Curriculum Assessment and Reporting Authority 2011). It is situated and connected with community (Overmars 2010). Learning evolves out of participation and engagement in a range of contexts—with the knowledge and its location situated in the daily lives of parents and children. This approach represents a positive view of the island community as containing rich cultural and cognitive resources with potential use in formal early childhood contexts.

## Literature review

Little is known about how Indigenous parents approach their children’s maths learning and how their approach interconnects with current Western mathematics teaching and learning in the early years. What is known is that Indigenous ways of knowing are described as the pedagogies or processes of learning in Indigenous cultures (Pember 2008). They are used to teach children in experiential ways that is, through modelling and storytelling that are deeply interconnected with family, community and culture.

### Indigenous ways of knowing and learning

Indigenous ways of knowing and learning are described as relational and interconnected because they are seen from a holistic perspective (Steinhauer 2002). They are about preparation for life rather than a measure of achievement and control. A relational nature of Indigenous ways of knowing is a stark contrast to Western ways of knowing which attempts to dissect, compartmentalize and measure nature to understand it (Overmars 2010, p. 90). This binary has impacted negatively on Indigenous parents and children because their ways of knowing have been depicted as inferior to Western ways of knowing. Indigenous ways of knowing are neither inferior nor superior to Western ways of knowing; they are a different perspective which needs to be acknowledged rather than trying to justify their inclusion in the realms of Western education (Brayboy and Castagno, 2008).

There are many differences between Indigenous and Western ways of knowing according to Hill (1999). One of the most telling is in Aboriginal thought a whole person consists of spirit, heart, mind and body—the capacity to see, feel, know, and do. Therefore, in the learning process, a whole person engages his or her physical, mental, emotional, and spiritual capacities in receiving data or information for the brain to process (Hill 1999, p. 100).

Hill (1999, p. 63) illustrates the capacity of the whole person to learn using a circle with four elements: to see, to feel, to know and to do. The first element involves the ability to see and have awareness “in relation, to self, family, community, nations, and the universe”. The ability to see is related to the second element, the ability to feel. With this element the child has to make decisions about struggling “personally with new information and problems that arise” (p. 63). Some of the information received may contradict assumptions, beliefs and attitudes of the child, thus causing struggle. The third element involves the mind and the ability to know. Here, the child resolves the contradictions to build new knowledge. The final element involves the body and the ability to do. Once the child has experienced awareness, struggle and building she or he can preserve a new sense of herself or himself. Children may learn best by seeing, feeling, knowing or doing which indicates if they are intuitive, emotional-relational, mental, or physically centred learners (Hill 1999). Although they might begin with awareness and move towards action, they can move around in the circle as they identify their preferred learning style. A model such as (Hill 1999) allows children, whose strengths may not be realised in a Western model, to develop and build confidence as learners. Overmars (2010) reinforces (Hill 1999) model about learning. Learning is gained through activities such as exploratory walks and field trips that allow children to explore their community in new ways or access parts of the community that are typically unavailable to them (Overmars 2010). These activities afford children strong connectedness with their community. If they know who they are and have a better understanding of their community and culture, they can develop pride and a greater motivation to learn within their communities.

### Involving parents in community and school education

There are specific advantages with adopting a holistic approach to involving parents and engaging children in learning whether it be in the community at school (Overmars 2010). Community based models of education are designed to involve parents and support children with their education and assist them with making the transition to formal schooling. Indigenous communities have unique resources in the form of Elders to support parents and children (Schissel and Wotherspoon 2003). Elders are often able to encourage and engage children in learning. They are able to pass on Indigenous knowledges to children that are specific to the community.

Community based education models are framed by the needs of the community, thus enabling parents and children to engage and focus on learning that has meaning and is contextualised with the community’s resources. Here, engagement refers to reflective involvement in deep understanding, valuing what is being done and actively participating in tasks. The result is a substantial sense of satisfaction and investment in learning. For children in the community, such experiences include Indigenous knowledge and ways of knowing (Schissel and Wotherspoon 2003). However, this does not suggest that Western ways of knowing are disregarded, as that would impact on how children interact in a dominant culture (Lee 2007; Overmars 2010). Overmars (2010) emphasises that the ability to “be biculturally competent is important to Indigenous peoples; thus the goal is not to live solely in an Indigenous framework (but) interconnect with Western framework” (p. 92) to include both ways of knowing to the benefit of children (see also (Bopp and Bopp 2001).

Community based models of education that draw on a community’s ways of knowing, practices and language, provide children with meaningful interactions in their communities. In doing so, children develop a strong interest in the community and are more likely to want to support the community in the future (Lee 2007). Through this model of education, relationships between children and communities are beneficial. It allows children to experience interconnectedness and build and strengthen relationships with community. Such experiences are invaluable with teaching children about Indigenous knowledge and Indigenous ways of knowing.

The following questions guided the project.

1. What mathematics do Torres Strait Islander parents know and understand?
2. How are these understandings connected with their community and daily practice?
3. How do their prior to school age children come to learn about mathematics?

## Methodology

A funds of knowledge approach was adopted as methodology in the project. This approach is most often associated with the work of Luis Moll (p. 132, 1992) although is also strongly associated with the work of Velez-Ibanez and Greenberg (1990). In their anthropological studies of United States Mexican Borderlands, Velez-Ibanez and Greenberg described the cultural resources and strategic information that households contained and which are needed to maintain well-being. Moll (1992) extended the funds of knowledge idea to education in the Borderlands in an attempt to disrupt the portrayal of communities as deficit and to transform teachers’ beliefs. Applying the funds of knowledge approach, he worked with teachers to explore how such funds could be brought to the classroom. Their studies found that households contained substantial cultural and cognitive resources with great potential for classroom instruction.

If one accepts the idea that funds of knowledge of mathematics are those that reflect the unique histories and culture of communities and which are historically and culturally accumulated, then the question arises: How are these knowledges and the learning of them holistic, connected with and situated in communities and the voices of the people? Here, I draw on the work of Lahn (2006) who describes the practice of giving fish. Giving a *sermaute* (share) of fish is a significant practice for Torres Strait Islander women. Whilst the choice of fishing companions can illustrate a range of relationships, for example, family and friendships, the “distribution of fish is not as flexible” (p. 301). With the division of caught fish, come the expectations to give a share to relatives as well as elderly neighbours. Distributing the fish is generally towards “ascending members of their own family and that of their husband” (p. 304). This emphasis reciprocates the earlier physical and social nurturance received by the individuals in this generation (in particular parents, aunties, mother’s brothers). These individuals are viewed as having nurtured them to adulthood, an idea communicated locally through expressions like *lugaut* (look after) and *gromape* (raised). … This ethic in fact extends to all older members of the community, who are seen as responsible in a more general sense for creating (nurturing) the physical and social community to which the younger generations now belong (p. 304).

Hence, women are expected to provide their family relations with fish of reasonable size and type. Through this process, the idea is to ensure that buckets have sufficient fish for each family, however, this does not mean that each bucket will have the same number of fish. Here, same is not based on amount rather it is the size of the family and ensuring that each family member receives food.

A funds of knowledge approach provides a powerful and rich way to learn about mathematical funds of knowledge that could be beneficial to teachers of children in the early years. The differences and lack of connection between maths as it occurs in daily life and in formal schooling are well documented in studies (see for example, Abreu 1995; Bishop 1988; Saxe 1991) that indicate that “adults and students are competent in performing mathematical tasks that they view as relevant” (Gonzalez et al. 2005, p. 257). All too often when children start school there is the leap to formal mathematics signs and symbols in classrooms that is not seen as relevant and connected to anything from their daily life experiences. With recent calls for changes in mathematics education (Australian Curriculum Assessment and Reporting Authority 2011) there is a need to capitalise on what children bring to the classroom as a consequence of the learning experiences they engage in before school through rich cultural practices.

### Location of the project

**Figure 1:**
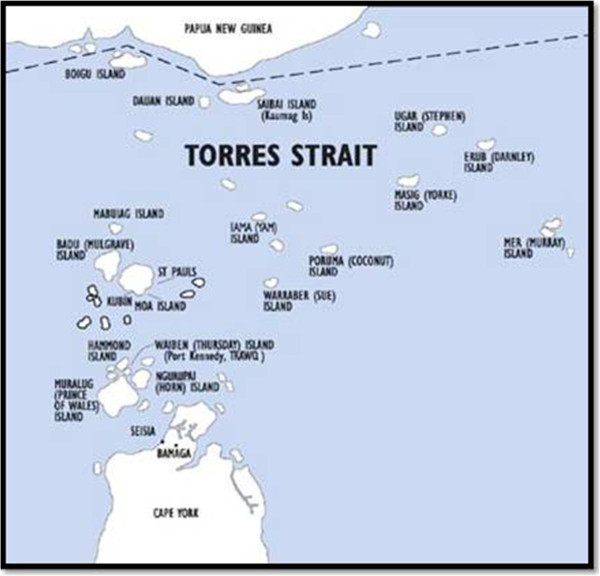
**Torres Strait regional authority map.**

The Torres Strait Islands consist of eighteen islands and two Northern Peninsula Area communities (Torres Strait Regional Authority 2010). They are geographically situated from the tip of Cape York north to the borders of Papua New Guinea and Indonesia and scattered over an area of 48, 000 square kilometres. There are five traditional island clusters in the Torres Straits: top western, western, central, eastern and inner islands (see Figure 1 Torres Strait Islander Regional Education Council 2011).

### Languages of the islands

Although I had visited the island on previous occasions as discussed in the background section earlier in this paper, I came from a background of speaking only one language, English, which was one of three languages spoken on the island or one of four spoken in the Torres Straits Islands.

Specific languages are spoken in Torres Strait Islander communities including Standard Australian English, Yumplatok (Creole), Kala Lagaw Ya (Mabuyag) and, Meriam Mir (Osborne 2009; Shnukal 2004). Kala Kawaw Ya (KKY) is understood to be a dialect of Kala Lagaw Ya (Osborne 2009). The traditional languages of the top western and western islands, Kala Lagaw Ya (KKY and Mabuyag) are understood to come from the mainland of Australia, with the eastern island language, Meriam Mir, emerging from Papua New Guinea. Yumplatok, identified as a modern language and stemming from colonisation, is derived from “meshing” both traditional languages and English thus creating a language in its own right (Osborne 2009; Shnukal 2004). This language is identified as unifying, that is, it is the one that everyone in the Torres Straits can speak, whereas the western traditional language speakers cannot speak and understand the eastern language speakers (Osborne 2009; Shnukal 2004).

### Participants

Twenty adults and eight children took part in the voluntary community consultation meeting. Three adults volunteered for the first workshop. One parent requested an individual workshop with her 3 year old son. All participants lived in the community. Three adults and one child are featured in this paper.

### Planning the community meeting

Community members who had a voluntary desire to participate were involved. Recent involvement with Indigenous communities taught me about the importance of meeting with community. There is little benefit derived from demanding that people should attend. When there is a sincere interest in engagement in learning in a community, relationships and trust can grow. Thus, what works is predicated on the assumption that if community can engage and identify with what is discussed, the more interest and enthusiasm is shown. In the lead-up to the community meeting, individual meetings were held with several people, including the Head of School Campus and the island Councillor to seek permission to hold the community meeting under the “Omei Tree”—Tree of Wisdom, a significant meeting place. This location was suggested by Denise, a senior community woman. I was also invited to take part in a radio interview about the project which was broadcasted live to the island community.

With support from Denise, and a parent, Cassie, from the community a paper-based flyer was delivered face-to-face to the homes of island parents to let them know about a proposed community meeting. At each stop around the community I was introduced by Denise and then asked to speak with the parents about the meeting. The content of the flyer was brief and aimed to provide succinct information about the meeting and its purpose. As per the flyer schedule, the meeting was held for one hour under the “Omei” Tree with community members invited to attend. A separate flyer was created for the workshop which was conducted in the Indigenous Knowledge Centre at a subsequent visit. It was distributed with the assistance of Paula, one of the parents who volunteered to assist with disseminating information to parents via word-of-mouth and email. Paula features later in this paper.

### Data collection techniques

The data collection techniques included asking questions that emerged as a conversation rather than as a research interview format. Using this method allowed for informal reflections in a relaxed context (Kovach 2010). Other data collection techniques included: digital photography, field notes and video/audio recording of a workshop. Digital photography as a non-written source of data allowed for the capturing of visual images that were central to the preliminary process and which served as a reminder for me (Stringer 2004). Field notes provided descriptions of places and events as they occurred and ongoing records of important elements of the setting and assisted with reporting and reflecting back over events. Video/audio recording served as a detailed reminder and which captured the participants’ knowledge and understandings verbatim (Stringer 2004). It also provided ongoing records of important elements of the setting. Each technique afforded the value of insight into the important preliminary planning of the project (Stringer 2004).

#### Data analysis technique

The data were analysed using illuminative experiences from conversations (Denzin and Lincoln 1998; Stringer 2004). This process allowed for re-presenting the participants’ experiences in decolonising ways and which maintained participants’ voices. In doing so, they captured where possible the meanings, emotions and ideas that can be applied to participants lives and understandings of their knowledges of mathematics. Illuminative experiences reveal features of experience not always apprehended in the normal course of events.

### Analysis and discussion of findings

In recent years, building on what communities bring to particular contexts and on their strengths has shown to be effective with engaging with communities (Gonzalez and Moll 2002). How does this occur? A way to engage the community was to start with something that was already familiar to them, and which then served as a basis for further discussion and learning (Gonzalez and Moll 2002).

#### The community meeting

At the commencement of the meeting, I introduced myself and explained who I was and where I was from. Some of the community members already knew me from the project work already conducted at the island school, living on the island and shopping at the local supermarket. I also explained some of my background and experiences as a matter of protocol and respect. By introducing myself to the community I provided information about my cultural location “so that connection can be made on political, cultural and social grounds and relations established” (Moreton-Robinson 2000, p. xv). This process allowed the community to locate me in the context of ancestry, where I was from and my family relations. That said, I began a discussion about mathematics, to gain an understanding of the community views of mathematics. This question invited conversations about mathematics, what they thought it was about, what they liked and disliked, where they used maths and who they learn it from. Their responses helped me to identify the maths they used in everyday life. This illuminative experience highlighted for me that I was looking at mathematics still largely as disembodied from the elements that are constitutive of daily life on the island.

**Figure 2:**
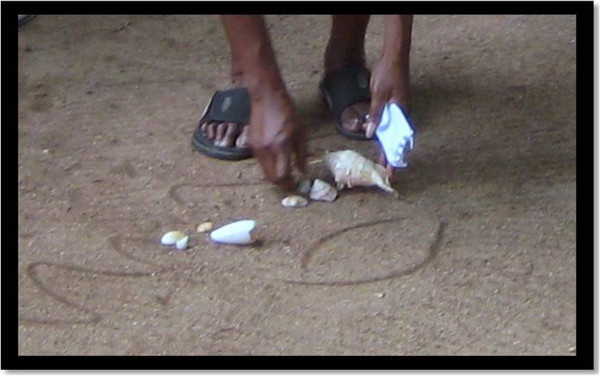
**Denise sorting shells.**

One example provided from the group was sorting shells. When I asked how they sort shells, Denise responded by taking the shells and showing how she sorted them and why (see Figure 2). Denise saw this task as relevant and connected with life in the community. She established the features of each of the sets of shells. The group then had to identify what criteria were used to sort the shells. If the criteria for membership to a group are vague, it is more challenging to decide whether the shells belong to a particular group. The group talked further amongst themselves.

Denise had established a criteria—edible and non-edible shell creatures. This criterion was relational with her ways of knowing. The group discussed what Denise had shown and explained that children learn about edible and non-edible shells when they were very young—one to two years of age and during times when families walked along the foreshores of the island and when fishing or playing in the water. Sorting activities assist with the promotion of understandings of grouping and similarities and diffences. Children and adults sort objects into groups using objects from their daily life experiences. They learn to identify sameness that defines the characteristics of groupings (Sousa 2008). The idea of creating and naming groups continues throughout life and is a way of creating and organising information and making connections with peoples’ experiences. Before young children can learn to count groups, they begin the process of defining a collection using the objects in their daily lives (Baroody and Benson 2001; Sousa 2008). Hence, they need experiences that have a rich variety of two- and three-dimensional objects. Noticing likenesses and differences among objects, children become aware of the features of different objects. They also become aware of grouping objects. Such an understanding paves the way for learning about partitioning.

My knowledge of sorting and defining the groups was different to how the community sorted and defined groups. This was an illuminative experience for me because I was using academically validated knowledge of mathematics which obscured “non-academic forms of mathematical practices” and processes used by the community (Gonzalez et al., 2005, p. 259). The sorting example reinforces that learning can be engaging and meaningful when it is situated within that which already exists— the culture, family and community. Gonzalez et al. (2005) elaborates this further by stating that maths is embedded in social knowledge and mediated through language and the activities of the community. It is not learned nor is it disembodied from its social meaning and context to become a linear process of dialog as happens within formal schooling. The knowledge of sorting edible and non-edible shell creatures was distributed among the group. It was a shared collective construction of mathematical knowledge embedded in culture. This experience of knowledge sharing was meaningful because it was associated with the environment and shared through daily living practices.

The meeting lasted for one hour. Towards the end, the members were asked if they wanted maths workshops for parents and children because this had been expressed in previous meetings with the Head of School Campus. Of importance was that the community needed time to network and discuss if they wanted me to return and work with parents and children on the island and if they identified that there were benefits for their community.

#### Workshop one

Building on what communities bring to particular contexts and on their strengths has shown to be effective when engaging with communities (Gonzalez and Moll 2002). This was evident from the community meeting. I was invited to do a workshop in the Indigenous Knowledge Centre on the island, a place of agency that permits and promotes engagement in a range of activities for the community (Taylor 2004). In doing so, I used the focus from the community meeting.

During the workshop, sorting and sharing emerged through conversation. Using items previously collected in the community, such as shells and seed pods, allowed for understandings of how these processes might be used to support their children’s learning.

The following excerpt shows how sorting, sharing and division as non-academic forms of mathematical practices emerged through conversation. Ailsa explained the process of giving fish. Ailsa drew on language that is associated with division. Of significance was that she explained how they involved children in such practices.

Ailsa Like you asked me what we do here, now when we come in with the fish and the ship in the community. . . we do with our kids and then wash and when we divide the fish among the families, like if I’ve got my 3 sisters and 2 brothers that I need to catch fish . . . with the fish, it doesn’t, we don’t all the bigger ones in this family and then the other sister get the small ones, we divide it quite evenly, like all the big fish in the basket, we get one each . . . And then we go down to the second size, even it up. . . When we do that, kids will stand there and say why don’t you put (indistinct) the question, so then we explain it to them. . . So we want it even.

**Figure 3:**
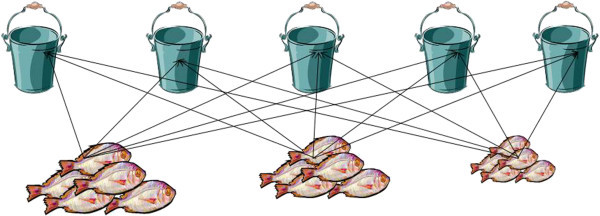
**Division as partitioning.**

In this excerpt four aspects were significant. The first was the use of the term “share” and that after the women had gone fishing, that “share” was for the community and their children. Second, the excerpt demonstrated how the parents’ and children’s social worlds were intertwined (Hill 1999). That is, Ailsa was thinking in conjunction with the resources and practices of her daily life which, in turn, were facilitated through the social relationships and contexts which constituted her life and that of the other parents and children to which she referred (Gonzalez et al. 2005). Through the holistic processes of doing, seeing and feeling as described by Hill (1999) the children learned the substance of sharing experiences that were used as opportunities for experimenting in other contexts and in doing so, built their knowledge of fair sharing via family activities and relations. Third, the actual practice required skills and knowledge in other areas, the division of fish among family relations—the social relationships involved in undertaking the practices and the deep connection with the process of fishing giving. Fourth, although I had no experience with fish giving and the rich cultural practices tied to this process, it was evident that the practice allowed for sharing/partitioning all the fish into parts as illustrated in Figure 3. Learning how to share and partition using hands-on objects such as fish, provides opportunities for developing the meaning of division. In school, division is presented as something that is done with signs and symbols which have little meaning for learners unlike the use of objects to share.

When partitioning, the number of groups is already known. How many objects must be placed in each group is not known. The ability to divide an object, such as an apple or a group of objects into equal parts is identified as critical to understanding the logical development of part-part, part-whole and whole-part relationships and notions of equality and inequality (Lamon 1996). This ability may also influence children’s understandings of mathematical topics such as measurement and geometry. Partitioning is a process that generates quantity and in doing so, builds understandings of rational numbers (Lamon 1996; Pothier and Sawada 1983). It is an activity that is intuitive and experienced-based. This process connects the construction of rational numbers (any number that can be made by dividing one by another) with children’s informal knowledge about fair sharing (Pothier and Sawada 1983). Unitizing however, is a cognitive process for coming to know and understand the amount of a given item or share before, during and after the sharing process.

Ailsa’s explanation provided insights into how the practice of fish giving allows for understandings of how the processes sharing/partitioning leads to equality, but not the equality as described by Lahn (2006) which was related to the status of family members. Ailsa’s explanation of how sharing occurred was not dissimilar to how it should be taught in school—using objects and starting with the whole. It also emphasised her ability to see and have awareness in relation to self, family, community and the environment (Hill 1999). It is these same practices that have the potential to be invisible through the trained eyes of formal education (Gonzalez et al. 2005). The maths involved in such practices, go “beyond facile constructs of social context and must take into account the deeply felt relationships of co-participants, the social relationships involved in undertaking the practices as well as the deep engagement of connection with a product, and not just a process” (p. 264). Ailsa explained the fish giving process—explaining that she had three sisters and two brothers that she needed to catch fish for and distribute evenly. The fish were first sorted into different sizes and then distributed using the process of sharing/partitioning. Partitioning was not found to be a possession that resided in Ailsa’s head as a fixed attribute or skill only known to her. Rather, partitioning was a practice, and giving fish created a context for the development and teaching of that practice as previously described. Gonzalez et al. (2005) argue that understanding maths is not simply about the possession of funds of knowledge in mathematical domains. The key point here is that such domains must be socially mediated into “productive knowledge in order to be meaningful” (p. 266).

What is also evident in the excerpt is that Ailsa and the women she referred to who were involved in the fish giving practice have the skills, connections and understandings with how the process works. It is up to the women to pass on this knowledge and support to their children because they are brought up this way and therefore it is what is expected (Lahn 2006). The excerpts revealed the process of fishing giving, who received fish and who taught the children, thus maintaining second generation and or third generation relations and practices.

#### Paula and Haile

In the following analysis, I provide a discussion of an interactive sequence between Paula and Haile who reside within the island community. The session was conducted and video-recorded in the Indigenous Knowledge Centre at Paula’s request and invitation. Paula was very enthusiastic about supporting her son Haile with learning about mathematics so as she could prepare him for school in the next two years. In preliminary conversations, Paula indicated that she wanted to know about algebra and how she could support Haile with his learning of the topic. She indicated that she found algebra very difficult to understand when she went to high school. I suggested that we might start with early algebra, such as repeating patterns because beginning with x’s and y’s does not develop deep arithmetical understandings. I explained that young children come to learn and recognise repeating patterns through actions such as jump, hop, jump, and hop and so on. Patterns are a way for children to recognise and organise their lives. In the early years, two particular pattern types are explored: repeating patterns and growing patterns. Repeating patterns are sequences of objects, pictures, or numbers that form a pattern because a section of them repeats, for example, OXOXOXOXOXO, repeating part: OX. What is critical is the ability to go from pattern to repeating part and repeating part to pattern (YuMi Deadly Centre 2012). They are used to find generalisations within the elements themselves (Warren and Cooper 2006). What comes next? Which part is repeating? Which part is missing?

The following excerpt provides an example of how Paula supported Haile, and in doing so, used shells placed on the table to show a repeating pattern. The shells were used because they were familiar to Haile, and in no way suggest cultural embeddedness. Indeed, in this excerpt other objects could have used—the shells were familiar, hence their use. Generally children explore patterns in a sequence: copy a pattern, continue the pattern, identify the elements repeating, complete the pattern, translate the pattern to a different medium (Warren and Cooper 2006).

I asked Haile if he was able to copy the pattern I had created using shells of different sizes. Where necessary, translations of Paula’s and Haile’s home language is translated to assist the reader. The excerpt below follows from that request.

Haile (Arranging shells left to right, passing each shell from his left hand to his right).

Paula (Hand relaxed and dropping behind Harry, says encouragingly) good boy. (After 14 seconds moves hand up and points to shells saying) big, the smaller one; speak, please can you speak? (Points to shells then relaxes arm to her side) big. . .

Haile (After thinking for 18 seconds says quietly in Yumplatok) big, smol (big = big; smol = small, little).

Paula (After watching and thinking for 21 seconds encourages Harry to speak asking) are you speaking?

Haile (Finishes pattern, points to each shell with his right index finger and says confidently) big, smol; big ane smol; big, smol, big, smol; big ane smol; big.

Paula (Says warmly) yes.

Haile (Smiles at Bron).

In this excerpt, Paula gently encouraged Haile to describe the elements in the pattern he had copied, big, smol, big, smol. She came to Haile’s aid because she could see what he had done with the shells. Of significance was Haile’s confidence to describe the pattern using his home language and gesture. In the next excerpt Haile was asked if he could make a pattern another way.

Haile (Combines the shells from his first pattern with the other shells on the table to the make a longer row of 19 shells. Fifty four seconds pass. Glances at camera finishes the long row, /?/speaks own language softly).

Paula (At the same time as Haile, straightens shells to her far left and speaks in own language –sounds like “em a tar kea”. A further 4 seconds pass). Spik. Spik a, spik nau (speak now).

Bron Okay. You show me.

Paula (Moves sideways shells upright and shows where Haile is to start and says) on this one, make em. (Pointing to first shell on the left which is too far away for Haile to touch. Moves her finger along the row, left to right till he can touch them for himself).

Haile (Moves his index finger above the first few shells, following his mother’s finger without touching saying) big, smol; big, smol. After 19 seconds thinking and pointing some confusion emerges as the two fingers are not pointing to the same shell. Also, the shells do not follow the ‘big, small’ pattern).

Paula (After watching Haile for 1 minute 23 seconds, stops pointing, pulls arm away, bent at elbow at her waist, between herself and Harry, ready to point if needed). After 9 seconds, moves arm behind Harry to show support.

Haile (Hands are clasped and is pointing with right index finger saying) big, smol; big, smol. After 1 minute 34 seconds, and not sounding confident, faulters saying) sm..ol., (glancing at camera). (Some of the shells are not following the ‘big, small’ pattern. Pauses for a second) big, big, smol..smol. (Finger is going to left as well as right, skipping shells). big and smol. (Speaking and pointing is now out of sync.)

Paula (Hand wavering behind Haile as she realises he is becoming unsure. Hand mes around Harry and she points to the 13^th^ shell saying) this one.

Haile (In time with Paula points to the 13^th^ shell also and continues more confidently saying) big, smol, big, smol . . . (whilst Paula points, his hands and finger moving along, following hers. Finishes with a triumphant) SMOL! (and grins).

In the above excerpt, Haile was asked to make a pattern another way, he responded to this request by incorporating more shells—nineteen, with the already made pattern, to make a longer pattern. He continued the pattern identifying the repeating elements. Haile was given time to think and engaged in deliberate attempts to solve the task posed drawing on familiar knowledge and placing out nineteen shells. Of significance was that Paula did not interrupt until Haile began to speak. Then, she gestured towards the shells encouraging Haile, who took over and pointed and touched each shell and uttered a corresponding description, “big, smol”.

Paula made the necessary adjustments to her gestures based on her observations of Haile. He showed how he could make a repeating pattern out of shells. Here, I refer to Vygotsky (1978) who states that pointing gestures were the first stage in the development of speech and that it is nothing more than an unsuccessful attempt to grasp. Over time a child’s movements “change with the act of pointing becoming a gesture of pointing for others and is understood by others as a gesture” (Ewing 2005, p. 44). Paula was influenced by Haile’s physical movements, gestures and facial expressions, all the while gently prompting and guiding him through the task. Where Haile became unsure, she gestured at the thirteenth shell with him. He then confidently continued to explain the pattern ending with a triumphant grin.

Of interest in the excerpts, was the willingness of Paula to engage with Haile and support him with learning how to copy and extend repeating patterns. Although there was minimal talk, the quiet moments, gestures, prompts and questions indicated that a deep and meaningful learning relationship was evident between the parent and child. Haile, as a 3 year old boy, showed great determination to achieve the task using the shells which were familiar to his environment, all the while being supported by Paula, his mother.

### Conclusion and implications

The illuminative experiences featured in this study highlight mathematics learning experiences that emerge from the daily practice of fish giving, Indigenous ways of knowing, and from using familiar objects. Indigenous ways of knowing become useful within the maths curriculum in schools as a means of stimulating and engaging students’ and their parents’ curiosity about their environment and their cultural practices in a context that is relevant to their lives. Although there is limited literature that focuses on how Indigenous parents teach their children mathematics, and considering the limited participant group in this study, it does illustrate that they do so using the cultural practices that are part of daily life. This perspective needs to be acknowledged. Through practices such as fish giving as described by Ailsa, knowledge is shared and exchanged. However, this process is not simply a process of giving fish. It is tied to culture, relationships and language.

Each family from which the women and children in this study come, the funds of knowledge accumulated and that form the basis of daily life, contains much of the previous generation’s repertoire of information and skills for living. These funds of knowledge are embedded in either historical or contemporary experiences of families. The funds and experiences are a “currency of exchange” (Moll 1992, p. 54) between generations and families that form the “cultural glue” (p. 54) that maintains cultural relations. This exchange and the idea of sharing and learning are embedded in the social knowledge of the women. It is mediated through the sharing experiences that the women perform and distribute among the group including the children.

This learning process is at odds with formal school mathematics, the dominant curriculum, which can obscure informal learning of mathematics and how children come to learn it through the practices of daily life. Thus, Indigenous ways of knowing can be depicted as inferior and not to the standard of Western ways of knowing mathematics, when it is about a different perspective which needs to be understood by educators if children are to be given opportunities to be educated in school in lasting ways that create future opportunities.

When children begin school, and where there is an unequal distribution of funds of knowledge and where materials and textbooks may be limited, drawing on the children’s cultural knowledge and understandings makes good sense (Browning-Aiken 2005). When children are provided with activities, such as the examples illuminated in this study, in their daily lives prior to schooling, a strong argument could be made that they should be much more closely linked when children commence formal learning of sorting, partitioning—division and early algebra.

There are several implications that are important to consider with this study: 1) the remoteness of the community and reasonable access to educational resources and support; 2) the extent that the community identified with the values of Indigenous ways of knowing and Western ways of knowing; 3) the extent that teachers in the community realise that mathematics in the community may be from a different perspectives and tied to deep cultural relations and practices and, 4) that parents may teach their children in experiential ways that are connected holistically with family, the community and environment. Each implication is tied inextricably to the other and could only be further understood over a longer time frame than the one provided in this study.

Finally, in evaluating the meeting, workshop and session as strategies for engaging with parents and their funds of knowledge of sorting, partitioning and early algebra the experience has revealed several themes that directly affect the nature of home—community relations—early years schooling and have the potential for improving educational achievements on the basis of more knowledge of pedagogical practices. How many teachers of young children are aware that this knowledge as it relates to sorting, partitioning and early algebra resides in the children’s daily experiences?
